# Unraveling the correlation between microbiota succession and metabolite changes in traditional Shanxi aged vinegar

**DOI:** 10.1038/s41598-017-09850-6

**Published:** 2017-08-23

**Authors:** Zhiqiang Nie, Yu Zheng, Sankuan Xie, Xianglong Zhang, Jia Song, Menglei Xia, Min Wang

**Affiliations:** 1 Key Laboratory of Industrial Fermentation Microbiology, Ministry of Education, College of Biotechnology, Tianjin University of Science and Technology, Tianjin, 300457 P. R. China; 2 Key Laboratory of Chemical Biology and Molecular Engineering, Ministry of Education, Institute of Biotechnology, Shanxi University, Taiyuan, 030006 P. R. China

## Abstract

Shanxi aged vinegar (SAV) is a well-known vinegar produced by traditional solid-state fermentation and has been used in China for thousands of years. However, how microorganisms and their metabolites change along with fermentation is unclear. Here, 454 high-throughput sequencing and denaturing gradient gel electrophoresis were used to investigate the composition of microbial community. Metabolites were further analyzed by gas chromatography–mass spectrometry and high–performance liquid chromatography. Results showed that the composition of bacterial community changed dramatically at different stages of fermentation. The bacterial genera (relative abundance > 0.1%) decreased from 17 in *daqu* (starter used in starch saccharification) to 2 at the 12th day of alcohol fernemtation (AF). 15 bacterial genera at the 1st day of acetic acid fermentation (AAF) decreased to 4 genera, involving *Acetobacter* (50.9%), *Lactobacillus* (47.9%), *Komagataeibacter* (formerly *Gluconacetobacter*, 0.7%) and *Propionibacterium* (0.1%) at the 7th day of AAF. The structure of fungal community was more homogeneous. *Saccharomyces* and *Saccharomycopsis* were predominant in AF and AAF. A total of 87 kinds of nonvolatile metabolites were detected. Canonical correspondence analysis showed a significant correlation between the microbiota succession and the formation of metabolites during the fermentation of SAV. This study provides detailed information for the fermentation mechanism of traditional SAV.

## Introduction

Shanxi aged vinegar (SAV), a well-known vinegar used in China for more than 3,000 years, is produced by traditional solid-state fermentation. The technique of traditional SAV involves the preparation of starter *daqu* (composed of barley and pea by spontaneous microbial growth on or in approximately 60% of the raw material), starch saccharification (SS) with starter *daqu*, alcoholic fermentation (AF), acetic acid fermentation (AAF), smoking, leaching, and aging^1, 2^. Different from other Chinese vinegars, SAV contains sorghum as the major ingredient. SS and AF are performed simultaneously after mixing with *daqu* powder and water. With added water, yeasts grow rapidly in AF. At the end of AF, production auxiliary materials involving pea, wheat bran, millet bran, and rice hull are added to increase porosity for oxygen uptake and heat discharge. The fermentation of SAV changes continuously from anaerobic fermentation to aerobic fermentation. Vinegar samples from the last batch of AAF are mixed with the alcoholic samples at the beginning of AAF and stirred manually every morning to maintain sufficient oxygen and improve acetic acid production. The whole fermentation process, including SS, AF, and AAF generally lasts approximately 20–30 days. After smoking and leaching, SAV must be aged for an additional one to eight years to bring out the vinegar’s unique taste and purity. According to the Chinese standard (GB/T 19777-2013, product of geographical indication - Shanxi extra aged vinegar), the traditional SAV contains more than 6 g/100 mL total acid and should be aged for more than 12 months.

Different environmental factors may lead to variation in microbial community composition. Several studies on microbial community composition in traditional Chinese vinegars have been reported, and results suggest that *Saccharomyces*, lactic acid bacteria (LAB) and acetic acid bacteria (AAB) are dominant functional microorganisms^2–7^. Focusing on microbial succession in single or double fermentation processes, the culture-independent techniques involving denaturing gradient gel electrophoresis (DGGE)^4, 5, 8–12^ and high-throughput sequencing^3, 13^ are applied in the microbiota analyses of vinegars. Several environmental factors, such as oxygen, heat, temperature, acidity, and salinity^14^, may influence the structure and biodiversity of a microbial community. Environmental factors, mainly oxygen and temperature, in SAV fermentation are empirically controlled to create a suitable fermentation environment for functional microorganisms that can produce necessary metabolites. Several studies on the volatile metabolites of SAV, Zhenjiang aromatic vinegar, and balsamic vinegars of Modena have been reported based on headspace solid-phase microextraction–gas chromatography (GC)–mass spectrometry(MS)^15–18^ and stir-bar sorptive extraction–GC–MS^19^. However, the correlations between metabolites and microorganisms during vinegar fermentation are still less known.

How communities assemble and which species can coexist in the same locale are central ecological questions^20^. Combining a classic filter model of community assembly with modern coexistence theory, the theoretical framework of community assembly and coexistence is proposed to unravel the selection of microbes and their interactions^20–22^. The direct adoption of models for community assembly developed for other organism groups is not evident when considering that the environmental condition changes^20^. The changes and succession of microbial community during vinegar fermentation have been investigated. Few studies focused on how metabolite changes with the variation of microbiota in vinegar fermentation. More recently, the relationship between bacterial dynamics and volatile metabolites in AAF of SAV has been reported, and the results show a strong correlation between the dynamics of the bacterial community and aromatic metabolites^23^. Increased scientific knowledge and improved vinegar manufacture technology are necessary and urgently needed to ensure higher standards of quality and safety in an expanding and increasingly diverse worldwide market^24^. The relationship between microbiota succession and metabolic activities, and competitive interactions along with the whole SAV fermentation process is rarely investigated. Therefore, the correlations between microbial succession and metabolite changes in the two fermentation stages (AF and AAF) should be further explored to unravel the fermentation mechanism of traditional SAV.

In this study, we tried to establish 1) how metabolites changed along with the succession of microbial community during the fermentation process of SAV, and 2) the correlation between metabolite changes and the variation of microbial community.

## Results

### Bacterial diversity and community succession in SAV fermentation

A total of 11 samples from SAV fermentation were chosen to analyze bacterial diversity and community succession by using 454 barcoded pyrosequencing. The V3–V5 regions of 16 S rRNA was amplified for 454 sequencing, and the results indicated that 477,223 raw sequencing reads with an average read length of 441 bp were obtained. A total of 297,126 high-quality sequences with an average read length of approximately 317 bp were obtained after low-quality and chimera sequences were removed (Supplementary Table S1). A total of 623 operational taxonomic units (OTUs) were obtained, and the abundance of OTU showed the genera abundances in SAV (Supplementary Table S1).

The α diversity metrics, including observed genera, Chao, ACE, Shannon, and Simpson indices, corresponded to the richness and the evenness of bacterial community in SAV (Supplementary Table S1). The indices involving observed genera, Chao and ACE showed that microbial richness increased in the first 7 days of AF and then decreased until the end of AF. In AAF, bacterial richness, which showed the same trend as AF, increased slightly in the first three days of AAF and then decreased. Shannon and Simpson indices showed that bacterial diversity in *daqu* was considerably higher than that in other samples. Bacterial diversity increased before 10 days of AF occurred, and bacterial diversity decreased afterwards. However, bacterial diversity decreased continuously until the end of AAF.

**Figure 1:**
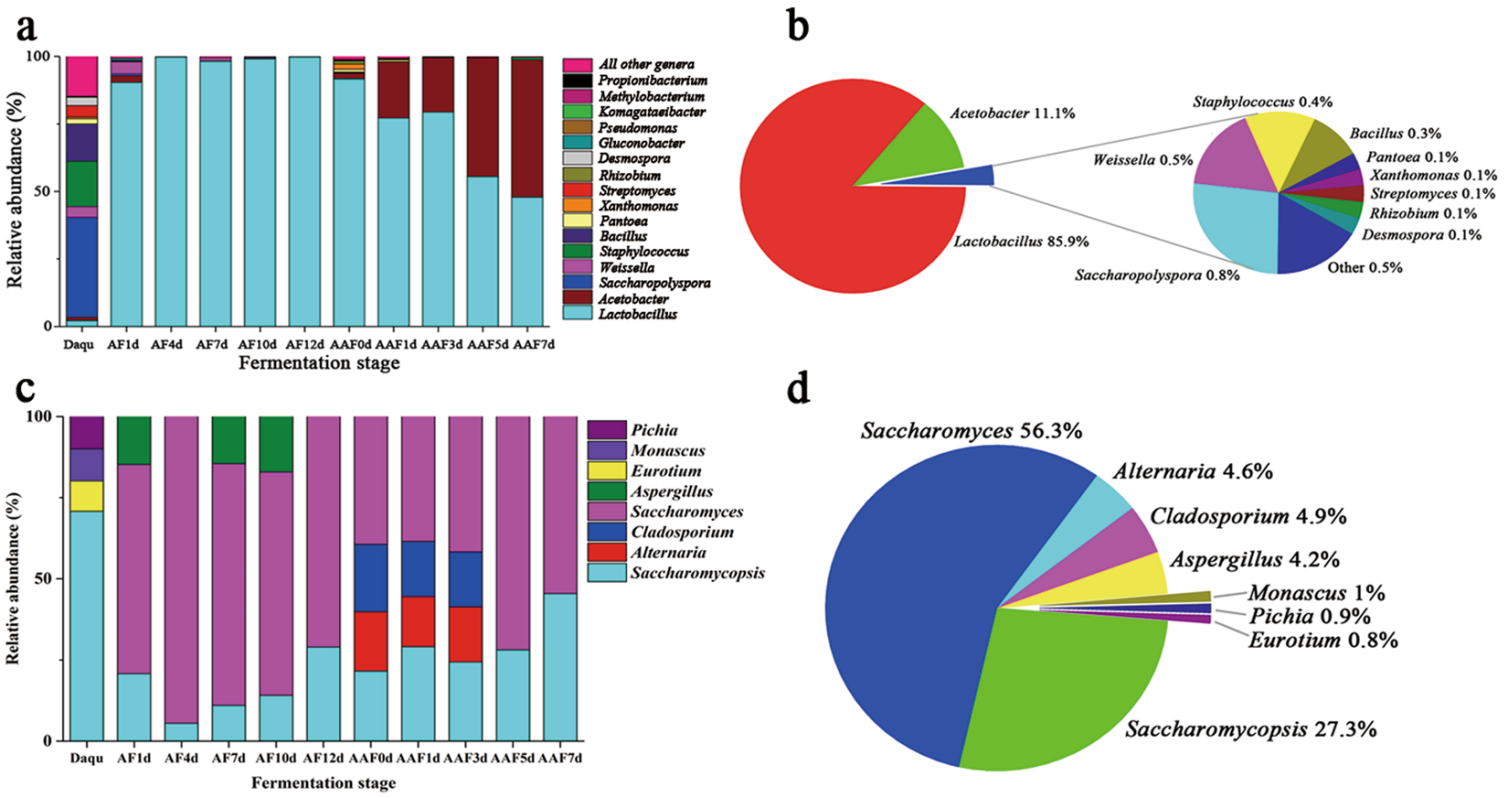
Succession of microbial community in different fermentation stages of Shanxi aged vinegar (SAV). (**a**) The distribution of major genera in the bacterial communities of different fermentation stages. (**b**) The total percentage of dominant bacterial genera in all specimens of SAV. (**c**) The distribution of major genera in the fungal communities of different fermentation stages. (**d**) The total percentage of dominant fungal bacterial genera in all specimens of SAV. *Daqu* represents the *daqu* specimen, AF1d–AF12d represent specimens from 1 day to 12 days of alcohol fermentation, and AAF0d–AAF7d represent specimens from 0 day to 7 days of acetic acid fermentation.

Bacterial community succession was investigated according to OTUs classified at a genus level. The changes and phylogenetic tree of different bacterial genera are shown in Fig. 1a and supplementary Fig. S1a. The distribution of bacterial genera with relative abundances of >0.1% in fermentation is shown in Fig. 1b. The results showed that more bacteria were found in *daqu* compared with other samples. The microbial community in *daqu* was dominated by 17 bacterial genera with a relative abundance of >0.1%, namely, *Saccharopolyspora* (37%), *Staphylococcus* (16.8%), *Bacillus* (13.8%), *Streptomyces* (4.2%), *Weissella* (4%), *Desmospora* (3.1%), *Lactobacillus* (2.2%), *Pantoea* (1.9%), *Acetobacter* (1.2%), *Xanthomonas* (0.7%), *Thermoactinomyces* (0.7%), *Saccharomonospora* (0.5%), *Pseudomonas* (0.3%), *Curtobacterium* (0.3%), *Brevibacterium* (0.2%), *Acinetobacter* (0.1%), and *Sphingomonas* (0.1%). Interestingly, bacterial community structure changed markedly once AF commenced. A majority of bacteria belonged to *Lactobacillus*, whose relative abundance was >98% during AF. At 1 day of AF, bacterial diversity rapidly decreased to eight genera, namely, *Lactobacillus* (90.4%), *Weissella* (4.5%), *Acetobacter* (2.5%), *Gluconobacter* (0.8%), *Saccharopolyspora* (0.7%), *Staphylococcus* (0.3%), *Pantoea* (0.1%), and *Corynebacterium* (0.1%). In late AF, *Lactobacillus* became the dominant bacteria, with a relative abundance of >99%, and other bacteria, such as *Acetobacter*, *Weissella*, *Saccharopolyspora*, and *Staphylococcus*, appeared with relative abundances of <0.4%. At 12 days of AF, only two bacterial genera (99.8% *Lactobacillus* and 0.1% *Acetobacter*) were detected. However, the number of genera increased once AAF commenced. We detected more than 15 bacterial genera (relative abundance > 0.1%), namely, *Lactobacillus* (91.7%), *Acetobacter* (2.2%), *Xanthomonas* (2%), *Rhizobium* (1.1%), *Pantoea* (1%), *Methylobacterium* (0.3%), *Pseudomonas* (0.2%), *Microbacterium* (0.2%), *Weissella* (0.1%), *Saccharopolyspora* (0.1%), *Staphylococcus* (0.1%), *Acinetobacter* (0.1%), *Sphingomonas* (0.1%), *Chryseobacterium* (0.1%), and *Pedobacter* (0.1%). A considerable number of bacteria disappeared as AAF progressed. *Acetobacter* increased rapidly from 20.8% (the 1st day) to 50.9% (the 7th day) and became dominant in AAF, in contrast to AF. On the contrary, *Lactobacillus* decreased from 77.2% (the 1st day) to 47.9% (the 7th day) in AAF. *Komagataeibacter* (formerly *Gluconacetobacter*) and *Propionibacterium* appeared at 5 days of AAF, and their abundances increased to 0.7% and 0.1%, respectively, at the end of AAF. The species of *Gluconacetobacter* involving *Gluconacetobacter europaeus*, *Gluconacetobacter oboediens*, *Gluconacetobacter nataicola* and *Gluconacetobacter intermedius* which are resistant against the higher concentration of acetic acid (>6%) have been recently reclassified into a novel described genus *Komagataeibacter* ^25, 26^.

**Figure 2:**
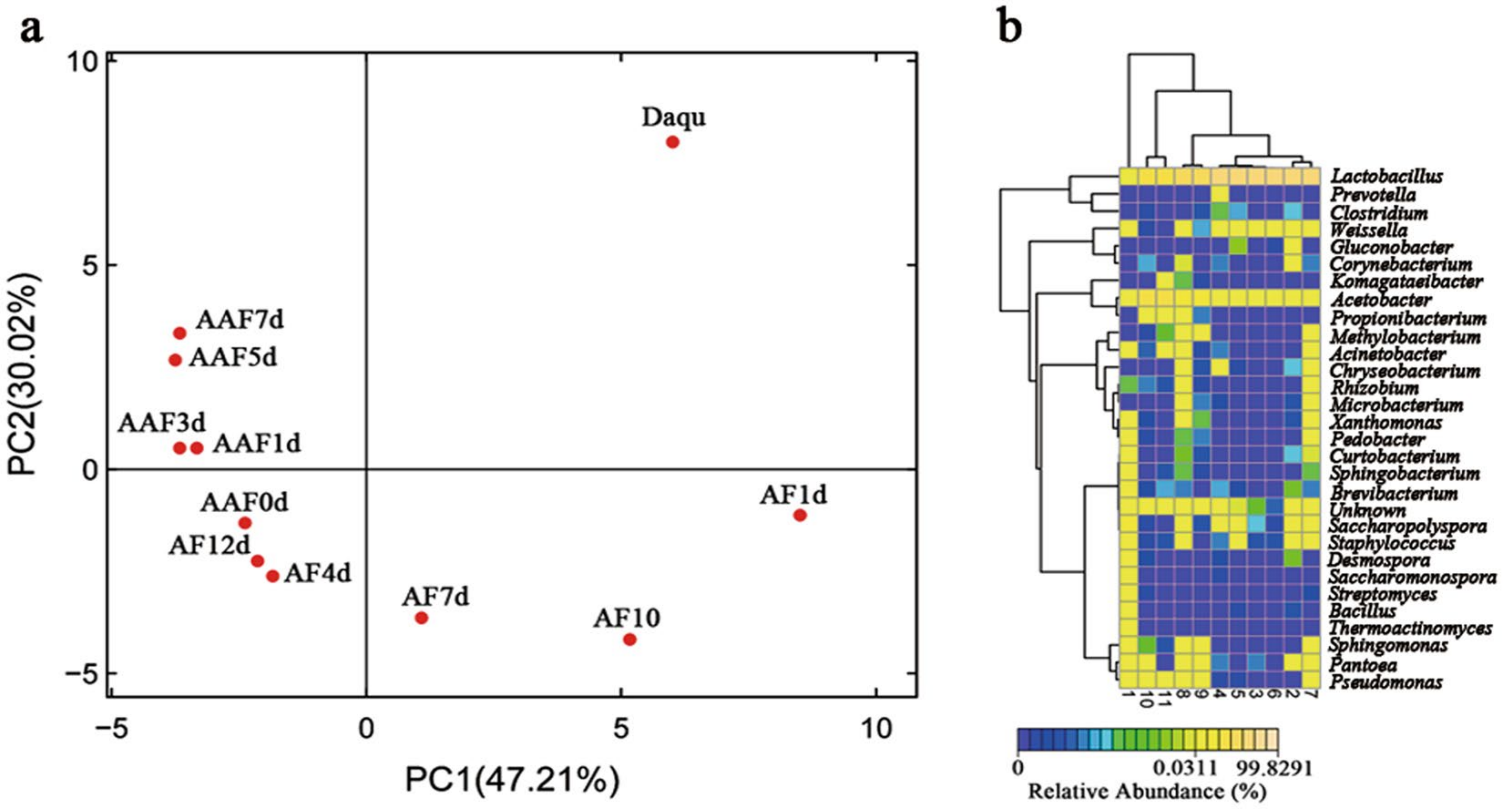
Principal component analysis (PCA) and hierarchical cluster analysis of bacterial community in the whole fermentation of SAV. (**a**) PCA score plot of 11 specimens based on metagenomes. The first two principal components (PC1 and PC2) can explain 77.23% of the data variance. (**b**) Changes in relative abundances of the dominant bacteria in 11 specimens. The color bar represents different abundances. *Daqu* represents the *daqu* specimen, AF1d–AF12d represent specimens from 1 day to 12 days of alcohol fermentation, and AAF0d–AAF7d represent specimens from 0 day to 7 days of acetic acid fermentation.

Differences in bacterial composition were further analyzed by PCA (Fig. 2a). The first axis (PC1) and the second axis (PC2) accounted for 47.21% and 30.02% of the variability, respectively. Bacterial composition in *daqu* was extremely different from other fermentation stages. Likewise, *jiulao* samples exhibited varying bacterial composition as AF progressed. The vinegar samples from the end of AAF are added as seeds at the beginning of AAF to accelerate the multiplication of AAB. Therefore, bacterial composition showed slight differences in AAF, but the bacterial compositions in the early (AAF1d–AAF3d) and later stages (AAF5d–AAF7d) were more similar. The changes in relative abundance of each bacterial genus were analyzed by hierarchical cluster analysis (Fig. 2b). The results show that *daqu* is more abundant in bacterial genera, but a majority of bacteria decreased in AF; eventually, bacterial abundances increased again in AAF.

### Diversity and succession of the fungal community in SAV fermentation

According to our previous report^12^, the diversity and the succession of the fungal community during fermentation were systematically investigated by DGGE (Fig. 1c, Supplementary Fig. S1b, Supplementary Fig. S2 and Supplementary Table S2). In summary, the microbial community throughout fermentation was dominated by eight fungal genera. The α diversity metrics indicate changes in fungal diversity (Supplementary Table S3). Shannon and Simpson indices show that the fungal diversity in *daqu* was the highest. Fungal diversity was relatively stable in AF, but this diversity decreased as AAF progressed. Figure 1d shows the distribution of fungal genera throughout fermentation. The fungal community was dominated by two genera, namely, *Saccharomyces* (85.9%) and *Saccharomycopsis* (11.1%). *Saccharomycopsis* was present throughout fermentation, and its relative abundance was more than 70% in *daqu*. During AF, the fungal community gradually decreased and was dominated by three genera, namely, *Saccharomyces*, *Saccharomycopsis*, and *Aspergillus*. *Saccharomyces* increased to 94.5% in the 4th day of AF and then decreased to 71.1% in the 12th day of AF. The relative abundance of *Saccharomyces* was >60% throughout AF. *Aspergillus* disappeared in 10 days of AF. *Saccharomycopsis*, *Cladosporium*, *Alternaria*, and *Saccharomyces* were all present during AAF. *Saccharomyces* and *Saccharomycopsis* became dominant in late (5 days to 7 days) AAF.

### Changes in metabolites throughout SAV fermentation

**Figure 3:**
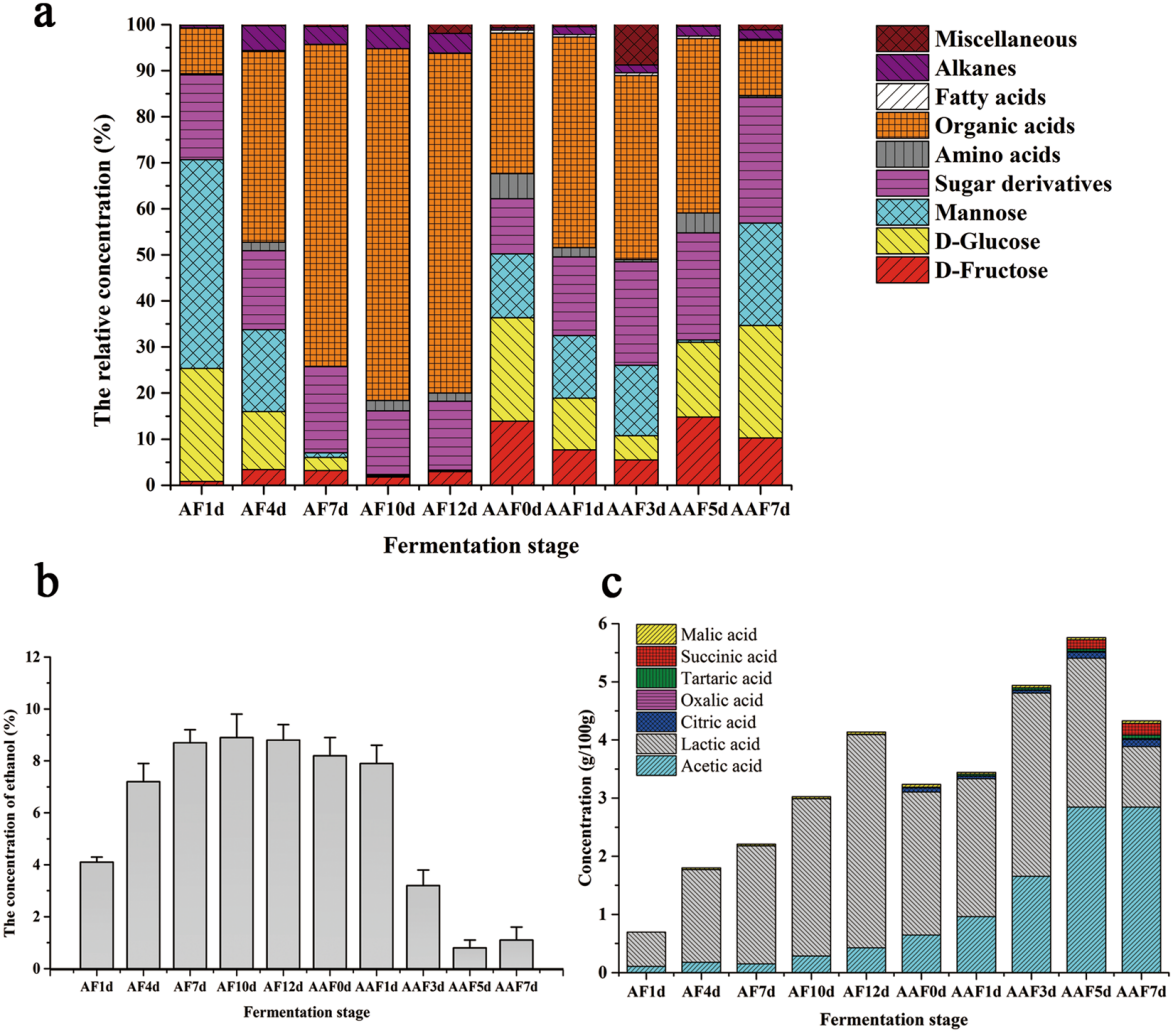
Distribution of metabolites in the whole fermentation of SAV. (**a**) Distribution of nonvolatile metabolites analyzed by GC–MS. Different colors represent different metabolites. All metabolites are divided into groups of sugar derivatives, alcohols, amino acids, and others, except for the relative higher abundances of glucose, fructose, and mannose. (**b**) Distribution of ethanol (%, v/v) analyzed by HPLC in different fermentation stages. (**c**) Distribution of seven dominant organic acids analyzed by HPLC in different fermentation stages. AF1d–AF12d represent specimens from 1 day to 12 days of alcohol fermentation, and AAF0d–AAF7d represent specimens from 0 day to 7 days of acetic acid fermentation.

A total of 87 kinds of nonvolatile metabolites during the whole fermentation of SAV were detected by GC–MS, including 12 amino acids, 19 organic acids, 5 fatty acids, 47 sugar and sugar derivatives, and 4 miscellaneous compounds (Supplementary Table S4). These metabolites were divided in groups of amino acids, alcohols, sugars and sugar derivatives, organic acids, fatty acids, alkanes, and other miscellaneous compounds (Fig. 3a). Figure 3a shows that the relative concentrations of sugars and sugar derivatives decreased along with AF. A total of 44 kinds of sugars and sugar derivatives were found in AF, whereas 42 kinds were detected in AAF (Supplementary Table S4). Among these sugars, D-glucose and mannose were present at more than 60% relative concentration in the *jiulao* specimen (AF1d), which represented the first day of AF. The relative higher concentration of sugars (glucose and mannose) may be mainly derived from the feedstock sorghum. The various hydrolytic enzymes in the starter *daqu* could hydrolyze the starch of sorghum into monosaccharides, such as glucose and mannose in starch saccharification. The *jiulao* specimen was treated directly by Trimethylsilyl (TMS) derivatization. Therefore, the relative concentrations of sugars and sugar derivatives were >75% after 1 day of AF. The concentrations of glucose and mannose decreased rapidly from 1 day to 6 days of AF. At 12 days of AF, the relative concentrations of glucose and mannose were 0.06% and 0.12%, respectively. These results suggest that sugars such as glucose and mannose are used by microorganisms compared with the growth of microbes (Fig. 1). The change in sugars in AAF was different from that in AF. The relative concentration of glucose and mannose in AAF1d was less than that in AF1d. The variation trend shows that the relative concentration of sugars decreases in the first 3 days of AAF and then increases until the last day of AAF. As a result of sampling from different ceramic urns, the metabolites in vinegar specimens show distinct differences. The instability of the relative concentrations of sugars (glucose and mannose) may also suggest the unsteadiness of traditional solid-state fermentation. A total of 12 kinds of amino acids and five kinds of alkanes were found in SAV. Although the relative concentrations of amino acid and alkane presented a growth trend in AF, the relative concentrations were both <3% at the end of AF and AAF.

The changes in ethanol in AF and AAF were analyzed by HPLC (Fig. 3b). The concentration of ethanol increased rapidly in AF and showed a decreasing trend in AAF. As shown in Fig. 1, yeasts including *Saccharomyces* and *Saccharomycopsis* grow rapidly along with AAF. Therefore, yeasts may contribute to the accumulation of ethanol in AF. A total of 19 kinds of organic acids were detected in AF and AAF. The organic acids also showed an increasing trend both in AF and AAF. To further understand the changes in organic acids, we analyzed seven kinds of dominant organic acids by HPLC (Fig. 3c). Five organic acids were detected in AF. Among these organic acids, lactic acid was the most abundant, with a concentration of 3.666 ± 0.975 g/100 g *jiulao* specimen at the end of AF. Lactic acid concentration gradually increased as AF progressed. Similarly, acetic acid concentration increased. Tartaric acid decreased gradually and disappeared at 5 days, whereas the two other organic acids (oxalic acid and malic acid) slightly increased as AF progressed. In AAF, acetic acid concentration increased rapidly as fermentation progressed. At 7 days, acetic acid was the dominant organic acid, with a concentration of 2.847 ± 0.187 g/100 g vinegar sample. Interestingly, lactic acid was also the dominant organic acid in AAF. While the concentration of lactic acid decreased to 1.039 ± 0.093 g/100 g sample at the end of AAF.

**Figure 4:**
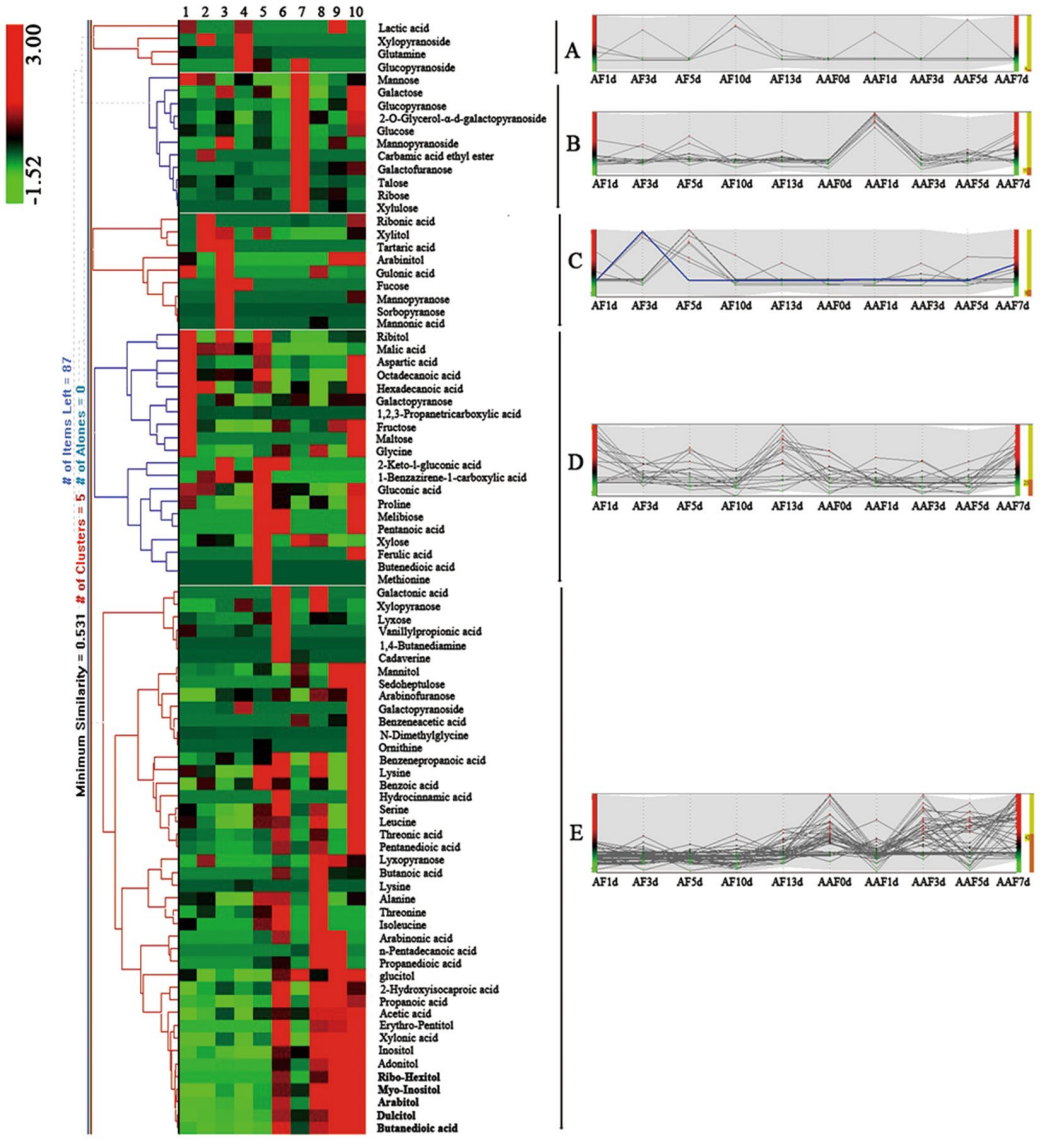
Hierarchial clustering analysis of 87 nonvolatile metabolites in different fermentation stages. Each row represents different nonvolatile compounds, and each column represents the fermentation time of SAV. Numbers 1–5 represent 1 day to 12 days of alcohol fermentation. Numbers 6–10 represent 0 day to 7 days of acetic acid fermentation. The color scale represents the relative concentration of different compounds. All metabolites are divided into five groups (A, B, C, D and E) according to their change trend.

The nonvolatile metabolites during the whole fermentation were clustered by using hierarchical cluster analysis (Fig. 4). Nonvolatile compounds were divided into five groups. The compounds in group A increased and dominated at the end of AF. Metabolites in group B, such as glucose, mannose, galactose, and mannopyranoside, showed a decreasing trend along with AF and AAF. The metabolites in group C increased to the top in the middle stage of AF and then disappeared. A total of 20 kinds of metabolites were included in group D, and their relative concentrations were higher in both the early and late stages of AF and AAF. The 43 kinds of nonvolatile compounds in group E showed a relatively higher concentration and an increasing trend in AAF. More than 50% of nonvolatile metabolites were obtained in AAF. Results indicate that AAF is an important fermentation stage of SAV for the formation of nonvolatile metabolites.

### Canonical correspondence analysis of the correlations between dominant microorganisms and metabolites

**Figure 5:**
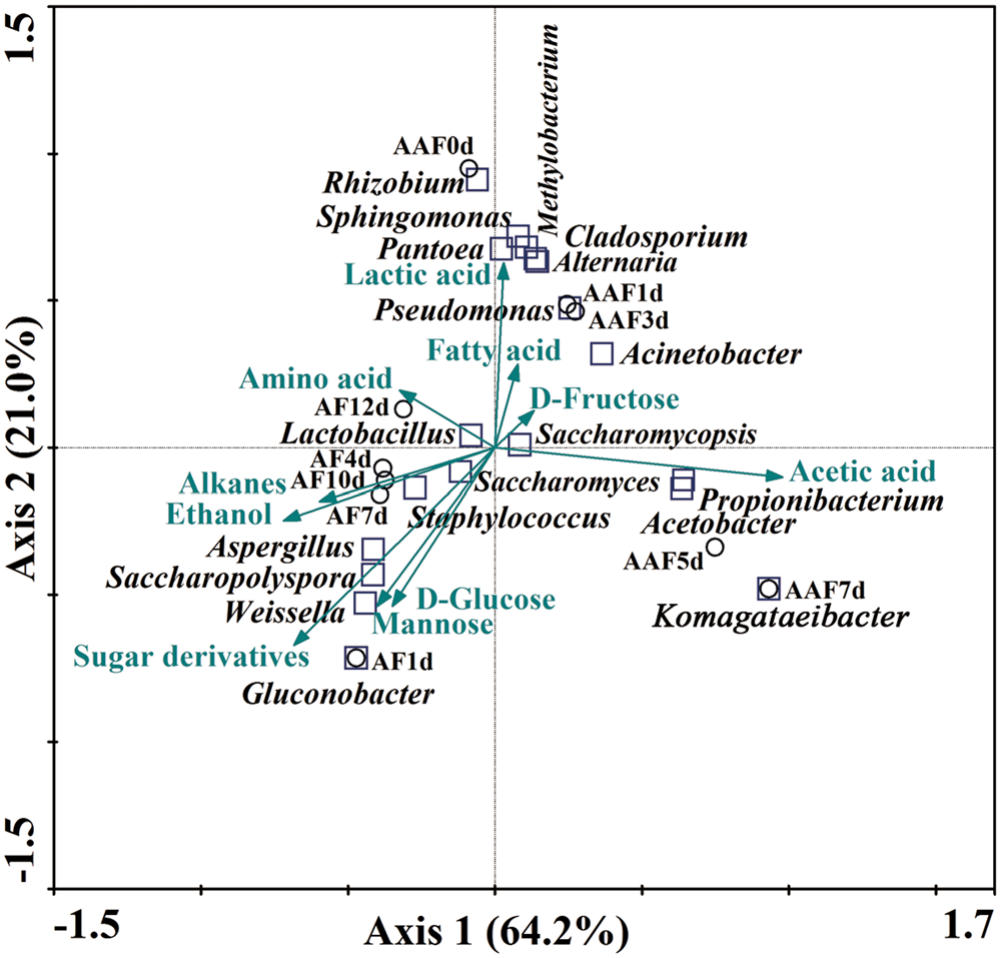
Canonical correspondence analysis of dominant microorganisms, fermentation time, and primary metabolites in SAV. Arrows represent different metabolites. Blocks represent dominant microorganisms. Circles represent different fermentation time. AF1d –AF12d represent 1 day to 12 days of alcohol fermentation, and AAF0d–AAF7d represent 0 day to 7 days of acetic acid fermentation. Axes 1 and 2 can explain 85.2% of the data variance.

Canonical correspondence analysis (CCA) could be used to investigate the correlations between environmental factors and microorganisms. Here, CCA was applied to understand the relationships between dominant microorganisms and metabolites based on the results of the present study (Fig. 5). Axes 1 and 2 can explain 85.2% of the data variance of the correlation between microorganisms and metabolites. The arrows represent the importance of metabolites. The distance between two points shows the significance of the correlation. For instance, the distance between *Saccharomyces* and AF10d is shorter than that between *Saccharomyces* and AF4d, suggesting that the amount of *Saccharomyces* is higher at 10 days of AF. The correlation between microorganisms and metabolites could be illustrated by sorting the vertical projection of point to the line of the arrow. The vertical projections are sorted from the origin to the direction of the arrow. The coordinate positions of vertical projections indicate the positive or negative correlation between microbes and metabolites. For instance, *Lactobacillus* shows the most significant positive correlation with lactic acid. The vertical projection of *Lactobacillus* on the line of lactic acid located above the origin of the coordinates indicates a positive correlation between lactic acid and *Lactobacillus*. Meanwhile, *Saccharomyces* shows a negative correlation with lactic acid. On the contrary, *Saccharomyces* shows a greater positive correlation with alcohols compared with other microbes, suggesting that this genera could contribute to the yield of alcohols. Ethanol is the dominant compound in alcohols; therefore, the statistical analysis performed in the present study confirmed that the genus *Saccharomyces* played an important role during the alcohol production (the first stage of vinegar production), which was also reported in previous studies^25, 27–29^.

The length of arrows determines the degree of importance. Figure 5 shows that D-glucose, mannose, sugar derivatives, and ethanol are more important in AF, whereas acetic acid is the most important factor in AAF. The results indicate that sugars and sugar derivatives present a positive correlation with the growth of microbes in AF. The length of ethanol indicates its importance as a factor in AF. Ethanol is the most important factor influencing microbial diversity in AF. In addition, ethanol shows a positive correlation on inhibiting the growth of numerous microorganisms. The influence of ethanol on *Lactobacillus* and *Saccharomyces* is weaker than that on *Aspergillus*, *Saccharopolyspora*, and *Weissella* in AF. *Saccharomyces* and *Lactobacillus* show higher stability with ethanol. Specimens of AAF show positive correlations with acetic acid, lactic acid, and fatty acid. The length of acetic acid indicates that acetic acid is the most significant factor in AAF, which is also confirmed by the previous report^27^. *Acetobacter*, *Komagataeibacter*, *Propionibacterium* and *Acinetobacter* show a significantly positive correlation with acetic acid. In accordance with previous experimental data^27^, *Acetobacter* and *Komagataeibacter* are the important acetic acid bacteria that are resistant to higher concentration of acetic acid. *Propionibacterium* and *Acinetobacter* could not secrete acetic acid; therefore, the positive correlation indicates relatively higher tolerance for acetic acid. Meanwhile, other microorganisms show less positive or negative correlation with acetic acid in AAF, indicating that the growths of the microorganisms are inhibited. A considerable number of microorganisms are disappeared in late (the 5th day to the 7th day) AAF because these microorganisms possess a low tolerance for acetic acid. Notably, lactic acid is another important metabolite which can inhibit the micrbioal multiplication both in AF and AAF. However, lactic acid influences microorganisms to a lower extent in AAF because its concentration decreases along with AAF. Lactic acid shows a positive correlation on inhibiting the growth of numerous microorganisms involving *Rhizobium*, *Sphingomonas*, *Pantoea*, *Pseudomonas*, *Methylobacterium*, *Cladosporium* and *Alternaria*. The influence of lactic acid on *Saccharomyces*, *Weissella*, *Lactobacillus*, *Gluconobacter*, *Acetobacter*, *Komagataeibacter* and *Propionibacterium* is weaker. In comparison, ethanol, acetic acid and lactic acid are important factors influencing the structures of microbial community during SAV fermentation.

### A theoretical model of correlations between microbiota succession and metabolite changes

**Figure 6:**
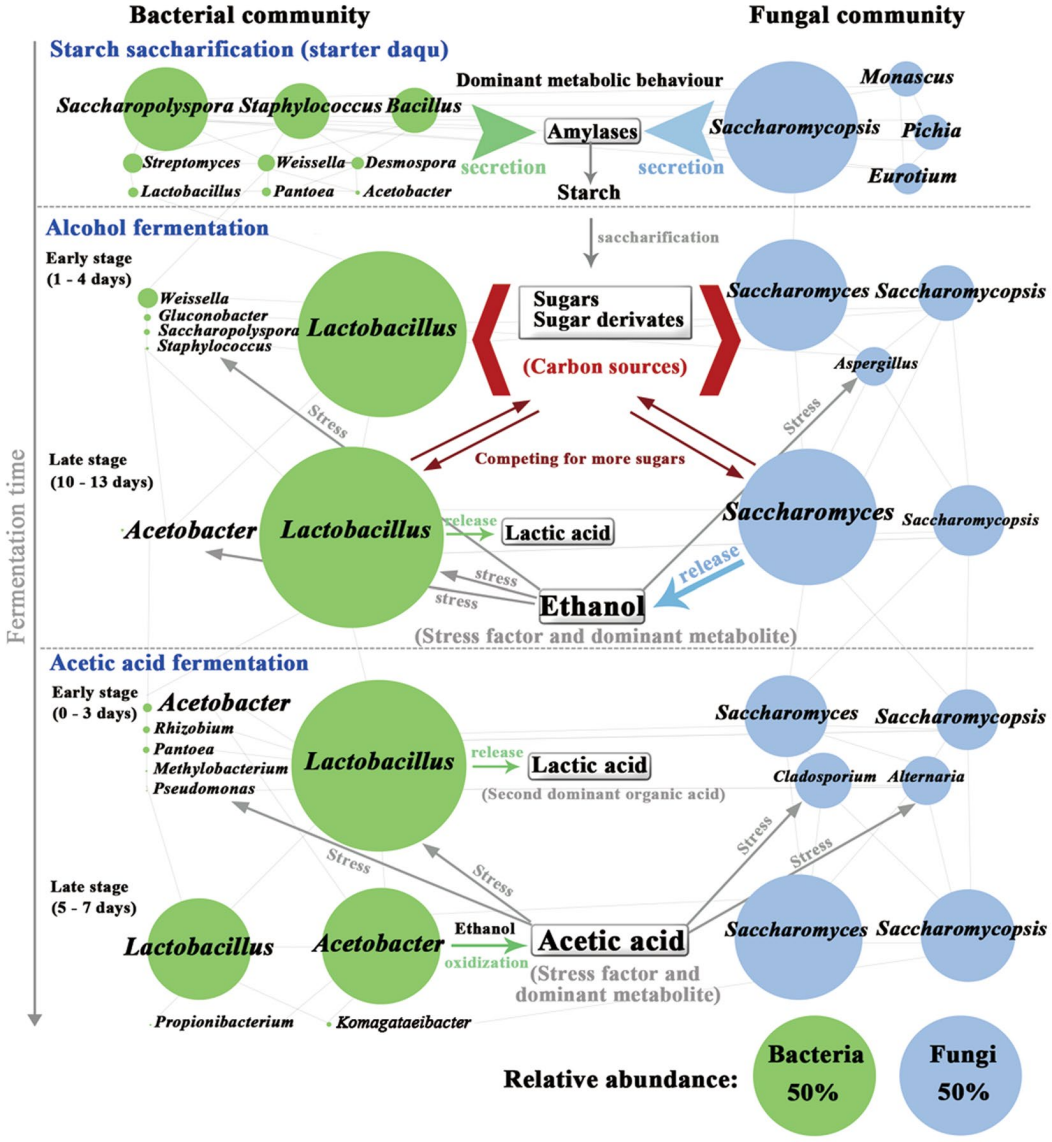
Schematic overview of the correlations between microbiota succession and nonvolatile metabolites in different SAV fermentation stages. The whole fermentation process is divided into five typical fermentation stages, including starch saccharification, alcohol fermentation (early stage and late stage), and acetic acid fermentation (early stage and late stage). The green circle represents the predominant bacteria, and the blue circle represents the predominant fungi. The area of each circle represents the relative abundances of each microorganism. The relative abundances of the circle area are listed in the bottom right. Arrows and lines represent the correlations between metabolites and microorganisms. The word “stress” represents the inhibition of different metabolites on microbial growth. The word “release” represents the secretion of metabolites from microorganisms.

A theoretical model was proposed to describe microbial community succession, metabolite changes, and competitive interaction in SAV fermentation (Fig. 6). Five typical SAV fermentation stages were described. The green circles and the blue circles represent the predominant bacteria and the predominant fungi, respectively (Fig. 6). The relative abundances of predominant microorganisms are represented by the circular area. Predominant microorganisms are connected with light gray lines, which form a large net that describes the relationship and correlation between microorganisms and metabolites. In SS, molds (*Monascus* and *Eurotium*), yeasts (*Saccharomycopsis* and *Pichia*), and certain bacteria (*Saccharopolyspora* and *Bacillus*) in *daqu* secrete amylases and transform starch into micromolecular sugars, such as glucose, mannose, and maltose, among others. AF occurs in an anoxic environment, which is suitable for the growth of facultative anaerobes, including yeasts and LAB. Therefore, *Saccharomyces*, *Saccharomycopsis*, *Lactobacillus*, and *Weissella* gradually became dominant in AF. However, microorganisms need to compete for resources, such as sugars. *Saccharomyces* could produce alcohol (mainly ethanol). Ethanol is an important stress factor that could inhibit the growth of a considerable number of microorganisms. *Saccharomyces* and *Saccharomycopsis* gradually become dominant in AF. *Lactobacillus*, which secretes lactic acid in late (the 10th day to the 13rd day) AF, also exhibit a higher tolerance for alcohols. AAF changes into aerobic fermentation, and numerous aerobic microorganisms grow rapidly in the early stage of AAF (0 day to 3 days). Among these microorganisms, AAB, particularly *Acetobacter* and *Komagataeibacter*, are predominant bacteria in AAF. *Acetobacter* can not only use micromolecular sugars as carbon sources but also oxidize ethanol to acetic acid^27^. According to the CCA results, acetic acid is an important stress factor inhibiting the enrichment of microorganisms in AAF. This result has also been supported by the the previous data^27^. A majority of bacteria and fungi are inhibited under the stress of acetic acid. As a result, *Acetobacter* and *Komagataeibacter* become the dominant microbes in AAF due to their higher tolerance to acetic acid^27^. Interestingly, *Lactobacillus* is also present throughout AAF. The relative abundance of *Lactobacillus* decreased to 47.9% in the late stage (5 days to 7 days) of AAF. *Lactobacillus* could be detected both in AF and AAF, suggesting that the genus may be domesticated to form a higher tolerance for ethanol and acetic acid.

## Discussion

The structure and composition of microbial community in traditional solid-state fermentation of SAV have been investigated previously in *daqu*, *jiulao*, and *cupei* samples, separately, using culture-dependent and culture-independent methods^2, 4, 7, 30, 31^. Wu *et al*. isolated cultivable microorganisms from *jiulao* and *cupei* of SAV^2^; their results show that 96% yeasts isolates are *Saccharomyces cerevisiae*, and only two isolates are *Pichia anomala*. We also detected *Saccharomyces* and *Pichia*. Differently, *Saccharomycopsis* is another dominant yeast that can not be detected by the culture-dependent method. *Alternaria*, *Cladosporium*, and *Eurotium* were all detected in SAV by the culture-independent method. However, several cultivable fungi, such as *Rhizopus* and *Candida*, could not be detected by DGGE, thereby demonstrating the limitation of DGGE^1^. Furthermore, numerous molds and yeasts, which can release various enzymes to accelerate starch saccharification, are found by culture-dependent methods^1, 2, 32^. The diversity and succession of fungal community should be further investigated by high-throughput sequencing.

LAB populations are also found as the dominant microorganisms in SAV^2, 4, 12^. Our results suggest that the relative abundances of LAB are more than 90% during AF and decrease to 49% at the end of AAF. These findings indicate that LAB may be an important functional microorganism for SAV. This result is beneficial for further investigation of the function of LAB. In addition to *Acetobacter*, *Komagataeibacter* and *Propionibacterium* were found as dominant acid-producing bacteria in SAV fermentation. These acid-producing bacteria may contribute flavor and aroma to SAV.

In comparing different Chinese vinegars, the diversity of microbial community is distinct for different brewing techniques, feedstock, and geographical location. For instance, Tianjin duliu mature vinegar (TDMV) from Tianjin, North China and Zhenjiang aromatic vinegar (ZAV) from Zhenjiang, East China also use the traditional solid-state fermentation^3, 5^. TDMV uses sticky rice as feedstock and a two-step solid-state acetic acid fermentation, in which only the *cupei* (the vinegar sample) in the top-half layer of the urn is stirred manually every morning. ZAV is made from glutinous rice, and its solid-state acetic acid fermentation generally lasts about 20 days. In the comparison of microbial compositions in AAF, a total of 13 bacterial genera (relative abundance > 0.1%) were found in TDMV based on Illumina sequencing of 16 S rRNA gene variable regions, whereas more than 15 bacterial genera (relative abundance > 0.1%) were detected in SAV. A total of 151 bacterial genera and 202 fungal genera were identified by metagenomics in ZAV^29^. *Lactobacillus* and *Acetobacter* are both dominant vinegar in the three kinds of vinegar, but the difference is that the abundance of *Acetobacter* in SAV is more than that in TDMV and ZAV.

The dynamics and diversity of microbial community in European vinegars using submerged fermentation have been described^10, 11, 13, 25, 33, 34^. Interestingly, AAB and LAB, which are also predominant microbiota in traditional Chinese vinegars^3, 12, 23, 28, 35, 36^, are also confirmed as two core microbiota in European vinegars. Trček *et al*.^25^ have analyzed diversity of microbiota invovlved in wine and organic apple cider submerged vinegar production using DHPLC and 16 S rRNA sequencing. Results show a very homogeneous bacterial structure during wine vinegar pruction but more heterogeneous during organic apple cider vinegar production. Differently, the AAB consortium is composed of *Acetobacter* and *Komagataeibacter* with the *Komagataeibacter* genus outcompeting the *Acetobacter* in all apple cider vinegar samples at the end of oxidation cycle. LAB consortium is composed of two dominating genera involving *Lactobacillus* and *Oenococcus* ^25^. However, only *Acetobacter* and *Lactobacillus* are two predominant genera at the end of AAF in SAV. The differences of core microbiota between Chinese and European vinegars may also come from different brewing techniques, feedstock and geographical location^3, 12, 25, 27, 28, 35^.

Bacterial dynamics and the changes of organic acids and volatile metabolites in AAF of SAV and ZAV have been reported^23, 28^. The results suggest a high correlation between the dynamics of bacterial community and metabolites, and our findings in the present study also confirm these results. Differently, we focused on the correlations between microbiota variation and metabolite changes both in AF and AAF of SAV. The bacterial compositions are slightly different in different vinegar companies of SAV. Numerous vinegar enterprises in Shanxi produce traditional SAV, and their techniques of traditional solid-state AAF are slightly different. For instance, the days of AAF in the present study are approximately 7 days, whereas those in the previous paper were about 19 days^23^. The previous study analyzed volatile metabolites in AAF^23^, whereas we focused on nonvolatile metabolites both in AF and AAF. Figure 3a shows that organic acids are important metabolites in SAV fermentation. The results of HPLC indicate that acetic acid and lactic acid are the dominant organic acids. The relative abundance of organic acids presents an increase trend in AF and decreases in AAF (Fig. 3a). HPLC analysis shows that the total concentration of organic acid showed an increase tend from AF to AAF. Lactic acid is a dominant organic acid in AF, but its abundance decreases in AAF. Acetic acid presents a stable and increasing trend in AAF. Succinic acid and citric acid also increased in 5 and 7 days of AAF. Other organic acids, such as lactic acid, malic acid, tartaric acid, and oxalic acid present a decreasing trend in the late AAF stage. Notably, the relative concentration of organic acids decreased at the 7th AAF day. Several metabolites, including amino acids, fatty acids, and alkanes, present an unstable and irregular change trend in AAF. One explanation for the instable change trend is the discontinuous sampling, and the other is the instability of traditional fermentation technique. This instability could also be found from the relative concentration of glucose in specimen AAF0d and AAF7d (Fig. 3a).

CCA results show that micromolecular sugars and sugar derivatives present a significant influence in AF. A large amount of sugars, such as glucose, fructose, and mannose in the *jiulao* specimen AF1d are not microbial metabolites and they may originate from the degradation of sorghum. The bacterial and fungal amylases in starter *daqu* can transform starches into oligosaccharides. α-Amylases, which randomly split α-(1 → 4)-linkages in starch, can be produced by bacteria and fungi, such as *Bacillus*, *Nocardiopsis*, *Lactobacillus*, *Aspergillus*, *Mucor*, and *Saccharomyces* genera, among others^37^. *Saccharomyces* grows faster than other anaerobes during AF. Ethanol production is a good ability of *Saccharomyces* ^38^. CCA results show that ethanol presents a significant correlation with yeasts. The function of ethanol changes in AAF; instead of being a stressor, ethanol functions as a substrate in AAF, which is already known in previous reports^39–41^. Additives (bran and rice hull and others) and daily manual stirring result in increased oxygen content; as a result, aerobic microorganisms, such as AAB, grow rapidly in the early AAF stage. Furthermore, the change in fermentation environment induced AAB to become the dominant microorganism, which could oxidize ethanol to acetic acid in AAF^39, 40^. Acetic acid is also found as a stress factor that could inhibit the growth of numerous microorganisms. Figure 5 shows that lactic acid have a higher influences in the late stages (12 days) of AF and the early stages (1 day to 3 days) of AAF. Lactic acid produced by LAB is the second dominant organic acid which can contribute to the fresh and sour taste of vinegar. However, the concentrations and influences of other metabolites such as amino acids and alkanes are relatively less than those of ethanol and acetic acid. These results indicate that *Saccharomyces* and *Acetobacter* are important functional microorganisms in AF and AAF, respectively. *Saccharomyces* and *Acetobacter* present high competitive ability and become dominant due to their wide habitat tolerance and good exploitation ability. The presence of *Saccharomycopsis* and *Lactobacillus* throughout fermentation indicates that these microorganisms gradually adapt to different environments and may also contribute to the formation of the SAV flavor and taste.

Microbial behaviors in a natural environment are difficult to investigate by high-throughput techniques because of the complex post-processing of highly abundant data^42^. The corresponding correlation between a specific metabolite with a specific microorganism is difficult to determine. Therefore, we used a group-to-group comparison to analyze the influence of metabolites on microbial community succession. The results suggest that metabolites present a significant correlation with microbiota succession and community assembly. However, the changes of volatile metabolites and their correlations with microbes in SAV fermentation need to be further investigated. Furthermore, a regulation study of a special functional gene maybe helpful in monitoring the production of functional metabolites. The function of *Lactobacillus* and its contribution to vinegar flavor need to be further investigated. The changes in microbiota and metabolites during fermentation are feasible to monitor, possibly leading the traditional technology to a controllable and mechanized production technology for traditional vinegars.

In this work, the microbiota succession and metabolite changes in AF and AAF of SAV were investigated systematically. In conclusion, a significant correlation between the succession of microbial community and the formation of metabolites during the whole fermentation process of SAV was confirmed by the statistical method and the rational analysis. This study provides detailed information for the fermentation mechanism of traditional SAV.

## Methods

### Sample collection

*Daqu*, alcoholic samples (called *jiulao* in Chinese), and vinegar samples (called *cupei* in Chinese) of SAV were collected from Qingxu, China. *Jiulao* samples (1, 4, 7, 10, and 12 days of AF) were periodically collected from the centre of the urn. *Cupei* samples (0, 1, 3, 5, and 7 days of AAF) were also collected at a depth of approximately 30 cm from the upper surface of the urn. Approximately 200 g of each sample was collected in triplicate and immediately stored in an ice box. Then, the samples were stored at −80 °C.

### Genomic DNA extraction

All samples were pretreated before DNA was extracted. Semi-liquid *jiulao* samples were centrifuged (8,000 × *g* for 10 min at 4 °C) to obtain pellets for DNA extraction. Approximately 1 g of solid sample was homogenized and ground by using liquid nitrogen. Subsequently, 500 mg of each sample was used to extract total genomic DNA according to a previously described method^3^.

### Amplification and 454 pyrosequencing

Bacterial 16 S rRNA genes (V3–V5 variable regions) were amplified from genomic DNA by using primers 341 F (5′-CCTACGGGNGGCWGCAG-3′) and 926 R (5′-CCGTCAATTCMTTTRAGT-3′), incorporating 454 FLX titanium adapters and a sample barcode sequence. Polymerase chain reaction (PCR) products were amplified and purified, as described previously^43^. Sequencing was performed using a 454 GS FLX titanium system (Roche, USA) at The Beijing Genomics Institute (BGI, Shenzhen, China).

### Processing and analysis of high-throughput sequencing data

16 S rRNA gene sequences were processed and modified as in previously described methods^44, 45^. Pyrosequencing data were processed using a data curation pipeline implemented in Mothur^46^. Sequencing reads were sorted to specific samples based on unique barcodes, and the barcodes were subsequently trimmed. Sequencing reads were removed if the reads were <200 bp or >1,000 bp, with a non-exact barcode match, more than two ambiguous bases (N), with more than eight consecutive identical bases, or with an average quality score <25. An NAST-based sequence aligner was used to align sequences to a custom reference based on SILVA alignment^44, 46^. Chimeric sequences were identified and quality-filtered. Chimera-free sequences were clustered in operational taxonomic units (OTUs) defined by 97% similarity by using a complete-linkage clustering tool. Representative sequences per OTU were classified according to previously described methods^44^. RDP naïve Bayesian rRNA classifier was used to hierarchically classify high-quality sequencing reads at the genus level with a confidence threshold of 80%^47^. Alpha diversity metrics, including Shannon−Weaver index, Chao1 estimate of richness, ACE index, and Simpson index, were calculated using Mothur (v. 1.31.2). The beta diversity of different samples was analyzed using QIIME (v. 1.50, http://qiime.org/index.html). The heat map of beta diversity was plotted using NMF package of R software (v2.15.3, http://www.r-project.org/). Principal component analysis (PCA) was conducted to investigate the relationship between vinegar samples and group microorganisms by using R software. The phylogenic tree was built by using neighbour-joining algorithm in MEGA software (v 6.0).

### DGGE analysis of fungal community

Fungal community was subjected to DGGE analysis according to a previously described method^3^. In this study, the D1 region of 26 S rDNA gene (primers NL1/LS2) was selected to analyze the structure of the fungal community. All bands were excised and reamplified. PCR products were purified and sequenced. Sequence analysis was carried out using BLAST algorithm in the GenBank nucleotide database (http://www.ncbi.nlm.nih.gov/blast/). DGGE profiles were assessed by Quantity One 4.6.2 (BioRad, Hercules, CA) to determine the information on band patterns.

### Analysis of nonvolatile metabolites and statistical analyses

Nonvolatile metabolites were detected by GC–MS. The semi-solid *jiulao* samples needed to be pre-frozen at −80 °C for 2 h and dried using a vacuum drying oven. The vinegar specimens were treated as follows. Two grams of pretreated *jiulao* or *cupei* specimens along with 10 mL of methanol and 1 mL of salicylic acid [1% (w/v) in methanol] as an internal standard were placed in a 50 mL vial. After being heated at 70 °C for 25 min and cooled at room temperature for 30 min, the mixture was added with 10 mL of distilled water and 5 mL of chloroform and treated with vortexing for 1 min. After being centrifuged twice at 12000 × g for 10 min, the aqueous fraction was extracted and then condensed to a final volume of 1 mL in a rotary vacuum evaporator. The moisture was completely removed by using a vacuum drying oven. TMS derivatization was performed to increase the volatility of nonvolatile compounds before GC–MS analysis according to a previously described method^48^. For TMS derivatization of the sugars and sugar derivatives, 300 µL of hexamethyldisilazane, 300 μL of trimethylchlorosilane (TMCS), and 400 μL of pyridine were added to a completely dehydrated specimen, and the mixture was heated at 85 °C for 50 min. The sample was then cooled at ambient temperature prior to GC–MS analysis. For other nonvolatile compounds, 300 µL of *bis*-(trimethylsilyl) trifluoroacetamide (BSTFA; Supelco, Bellefonte, PA, USA) containing 1% TMCS and 300 μL of acetonitrile were added to the dehydrated specimen and then heated at 70 °C for 40 min followed by cooling to ambient temperature. A HP-5 column (30 m length × 250 μm internal diameter × 0.25 μm film thickness; Agilent, USA) attached to an Agilent 6890 N series gas chromatograph that was connected to a 5973 mass selective detector (Agilent, USA) was employed. The temperature levels of the injector and the detector transfer line were 200 °C and 250 °C, respectively. The oven temperature was maintained at 50 °C for 2 min, increased to 150 °C at a rate of 4 °C·min^−1^ and maintained for 5 min, increased to 220 °C (2 °C·min^−1^) and maintained for 3 min, and increased to 280 °C at a rate of 3 °C·min^−1^ and maintained for 4 min. Helium was used as a carrier gas at a constant flow rate of 0.8 mL·min^−1^, and mass spectra were obtained at 70 eV through the electron ionization method. The injection volume was 1.0 μL. Nonvolatile compounds were identified by comparing their retention times and mass spectra with those of authentic standards. The concentrations of nonvolatile compounds were calculated by comparing the peak areas with those of the internal standard compounds. The quantitative data were the mean values of triplicate measurements.

The ethanol changes in the whole fermentation were analyzed by HPLC according to a previously reported method^12^. Seven kinds of predominant organic acids (acetic acid, lactic acid, malic acid, tartaric acid, oxalic acid, succinic acid, and citric acid) in SAV were further analyzed by HPLC according to a previously described method^49^. Standard substances of organic acids were purchased from Sigma-Aldrich (USA). HIERARCHICAL CLUSTERING EXPLORER 3.5 software (www.cs.umd.edu/hcil/hce/) was used to perform hierarchical clustering of nonvolatile metabolites with a complete linkage method by using the Pearson correlation distance. Canonical correspondence analysis (CCA) was performed using Canoco for Windows v4.5 (Wageningen UR, Netherlands) to investigate the correlations between microbial genera and metabolites. All data should be normalized by SPSS software to eliminate the influence of different dimensions before using in CCA.

## Electronic supplementary material

Supplementary information

## References

[1] Chen, F. S., Li, L., Qu, J. & Cheng, C. X. In *Vinegars of the World* (eds L. Solieri & P. Giudici) Ch. 15, 243–259 (Springer, 2009).

[2] Wu JJ, Ma YK, Zhang FF, Chen FS (2012). Biodiversity of yeasts, lactic acid bacteria and acetic acid bacteria in the fermentation of “Shanxi aged vinegar”, a traditional Chinese vinegar. Food Microbiol..

[3] Nie Z (2013). Exploring microbial succession and diversity during solid-state fermentation of Tianjin duliu mature vinegar. Bioresource Technol..

[4] Wu JJ, Ma YK, Zhang FF, Chen FS (2012). Culture-dependent and culture-independent analysis of lactic acid bacteria from Shanxi aged vinegar. Ann. Microbiol..

[5] Xu W (2011). Monitoring the microbial community during solid-state acetic acid fermentation of Zhenjiang aromatic vinegar. Food Microbiol..

[6] Wu JJ, Gullo M, Chen FS, Giudici P (2010). Diversity of Acetobacter pasteurianus Strains Isolated From Solid-State Fermentation of Cereal Vinegars. Curr. Microbiol..

[7] Hao L, Yang N, Hu HP, Bai RH, Qin N (2008). Isolation and identification of acetic acid bacteria from Shanxi superior mature vinegar in China. Am. Lab..

[8] Mamlouk D, Hidalgo C, Torija MJ, Gullo M (2011). Evaluation and optimisation of bacterial genomic DNA extraction for no-culture techniques applied to vinegars. Food Microbiol..

[9] Haruta S (2006). Succession of bacterial and fungal communities during a traditional pot fermentation of rice vinegar assessed by PCR-mediated denaturing gradient gel electrophoresis. Int. J. Food Microbiol..

[10] Gullo M, De Vero L, Giudici P (2009). Succession of Selected Strains of Acetobacter pasteurianus and Other Acetic Acid Bacteria in Traditional Balsamic Vinegar. Appl. Environ. Microb..

[11] De Vero L (2006). Application of denaturing gradient gel electrophoresis (DGGE) analysis to evaluate acetic acid bacteria in traditional balsamic vinegar. Food Microbiol..

[12] Nie Z, Zheng Y, Du H, Xie S, Wang M (2015). Dynamics and diversity of microbial community succession in traditional fermentation of Shanxi aged vinegar. Food Microbiol..

[13] Valera MJ, Torija MJ, Mas A, Mateo E (2015). Acetic acid bacteria from biofilm of strawberry vinegar visualized by microscopy and detected by complementing culture-dependent and culture-independent techniques. Food Microbiol..

[14] Steudel B (2012). Biodiversity effects on ecosystem functioning change along environmental stress gradients. Ecol. lett..

[15] Chen T, Gui Q, Shi JJ, Zhang XY, Chen FS (2013). Analysis of variation of main components during aging process of Shanxi Aged Vinegar. Acetic Acid Bacteria.

[16] Lu ZM (2011). Recovery of aroma compounds from Zhenjiang aromatic vinegar by supercritical fluid extraction. Int. J. Food Sci. Tech..

[17] Cirlini M, Caligiani A, Palla L, Palla G (2011). HS-SPME/GC-MS and chemometrics for the classification of Balsamic Vinegars of Modena of different maturation and ageing. Food Chem..

[18] Zhu H, Zhu J, Wang L, Li Z (2016). Development of a SPME-GC-MS method for the determination of volatile compounds in Shanxi aged vinegar and its analytical characterization by aroma wheel. J. Food Sci. Technol..

[19] Marrufo-Curtido A (2012). Characterization and differentiation of high quality vinegars by stir bar sorptive extraction coupled to gas chromatography-mass spectrometry (SBSE–GC–MS). LWT - Food Sci. Technol..

[20] Valyi K, Mardhiah U, Rillig MC, Hempel S (2016). Community assembly and coexistence in communities of arbuscular mycorrhizal fungi. Isme J..

[21] HilleRisLambers J, Adler PB, Harpole WS, Levine JM, Mayfield MM (2012). Rethinking Community Assembly through the Lens of Coexistence Theory. Annu. Rev. Ecol. Evol. S..

[22] Chesson P (2000). Mechanisms of maintenance of species diversity. Annu. Rev. Ecol. Syst..

[23] Li S, Li P, Liu X, Luo L, Lin W (2016). Bacterial dynamics and metabolite changes in solid-state acetic acid fermentation of Shanxi aged vinegar. Appl. Microbiol. Biotechnol..

[24] Solieri, L. & Giudici, P. *Vinegars of the World*. (Springer, 2009).

[25] Trček J, Mahnič A, Rupnik M (2016). Diversity of the microbiota involved in wine and organic apple cider submerged vinegar production as revealed by DHPLC analysis and next-generation sequencing. Int. J. Food Microbiol..

[26] Yamada Y (2012). Description of *Komagataeibacter* gen. nov., with proposals of new combinations (Acetobacteraceae). *J*. Gen. Appl. Microbiol..

[27] Trček J, Mira NP, Jarboe LR (2015). Adaptation and tolerance of bacteria against acetic acid. Appl. Microbiol. Biotechnol..

[28] Wu LH (2017). Metagenomics reveals flavour metabolic network of cereal vinegar microbiota. Food Microbiol..

[29] Wang ZM, Lu ZM, Shi JS, Xu ZH (2016). Exploring flavour-producing core microbiota in multispecies solid-state fermentation of traditional Chinese vinegar. Sci. Rep..

[30] Wu, J. H., Hao, L. & Bai, R. H. Ecological distribution of microbe in Shanxi super vinegar Daqu. *J. Shanxi Agricultur. Univer*. **24**, 279–282 (2004).

[31] Hu, H. P. & Hao, L. Separation and identification of superior Acetobacter acetic from Shanxi super-mature vinegar. *J. Shanxi Agricultur. Univer*. **24**, 283-285 (in Chinese) (2004).

[32] Liu DR (2004). Chinese vinegar and its solid-state fermentation process. Food Rev. Int..

[33] Vegas C (2010). Population dynamics of acetic acid bacteria during traditional wine vinegar production. Int. J. Food Microbiol..

[34] Ilabaca C, Navarrete P, Mardones P, Romero J, Mas A (2008). Application of culture culture-independent molecular biology based methods to evaluate acetic acid bacteria diversity during vinegar processing. Int. J. Food Microbiol..

[35] Wang ZM (2015). Batch-to-batch uniformity of bacterial community succession and flavor formation in the fermentation of Zhenjiang aromatic vinegar. Food Microbiol..

[36] Peng Q, Yang Y, Guo Y, Han Y (2015). Analysis of Bacterial Diversity During Acetic Acid Fermentation of Tianjin Duliu Aged Vinegar by 454 Pyrosequencing. Curr. Microbiol..

[37] Souza PM, Magalhaes PO (2010). Application of microbial α-amylase in industry - a review. Braz. J. Microbiol..

[38] Perrone B, Giacosa S, Rolle L, Cocolin L, Rantsiou K (2013). Investigation of the dominance behavior of Saccharomyces cerevisiae strains during wine fermentation. Int. J. Food Microbiol..

[39] Trček J, Toyama H, Czuba J, Misiewicz A, Matsushita K (2006). Correlation between acetic acid resistance and characteristics of PQQ-dependent ADH in acetic acid bacteria. Appl. Microbiol. Biotechnol..

[40] Matsushita K, Takaki Y, Shinagawa E, Ameyama M, Adachi O (1992). Ethanol Oxidase Respiratory Chain of Acetic Acid Bacteria. Reactivity with Ubiquinone of Pyrroloquinoline Quinone-dependent Alcohol Dehydrogenases Purified from *Acetobacter aceti* and *Gluconohacter suhoxydans*. Biosci. Biotechnol. Biochem..

[41] Thurner C, Vela C, Thöny-Meyer L, Meile L, Teuber M (1997). Biochemical and genetic characterization of the acetaldehyde dehydrogenase complex from *Acetobacter europaeus*. Arch. Microbiol..

[42] De Roy K, Marzorati M, Van den Abbeele P, Van de Wiele T, Boon N (2014). Synthetic microbial ecosystems: an exciting tool to understand and apply microbial communities. Environ. Microbiol..

[43] Zhang X (2013). Human gut microbiota changes reveal the progression of glucose intolerance. Plos One.

[44] Kostic AD (2012). Genomic analysis identifies association of Fusobacterium with colorectal carcinoma. Genome Res..

[45] Jeong SH, Jung JY, Lee SH, Jin HM, Jeon CO (2013). Microbial succession and metabolite changes during fermentation of dongchimi, traditional Korean watery kimchi. Int. J. Food Microbiol..

[46] Schloss PD (2009). Introducing mothur: open-source, platform-independent, community-supported software for describing and comparing microbial communities. Appl. Environ. Microbiol..

[47] Wang Q, Garrity GM, Tiedje JM, Cole JR (2007). Naive Bayesian classifier for rapid assignment of rRNA sequences into the new bacterial taxonomy. Appl. Environ. Microbiol..

[48] Namgung HJ (2010). Metabolite profiling of doenjang, fermented soybean paste, during fermentation. J. Sci. Food Agr..

[49] Eyéghé-Bickong HA, Alexandersson EO, Gouws LM, Young PR, Vivier MA (2012). Optimisation of an HPLC method for the simultaneous quantification of the major sugars and organic acids in grapevine berries. J. Chromatogr. B.

