# Supplementary material for: Unraveling the correlation between microbiota succession and metabolite changes in traditional Shanxi aged vinegar
Source: Sci Rep. 2017 Aug 23;7:9240. doi: 10.1038/s41598-017-09850-6 (PMC5569029; doi:10.1038/s41598-017-09850-6)
Supplement: Supplementary file 1 — Supplementary information [file 41598_2017_9850_MOESM1_ESM.pdf]

Unraveling the correlation between microbiota succession and metabolite changes in traditional Shanxi aged vinegar

Zhiqiang Nie[1, 2], Yu Zheng[1], Sankuan Xie[1], Xianglong Zhang[1], Jia Song[1], Menglei Xia & Min Wang[1, *]

[1]Key Laboratory of Industrial Fermentation Microbiology, Ministry of Education, College of Biotechnology, Tianjin University of Science and Technology, Tianjin 300457, P. R. China

[2]Key Laboratory of Chemical Biology and Molecular Engineering, Ministry of Education, Institute of Biotechnology, Shanxi University, Taiyuan 030006, P. R. China

*Correspondence: Min Wang, Key Laboratory of Industrial Fermentation Microbiology, Ministry of Education, College of Biotechnology, Tianjin University of Science and Technology, Tianjin 300457, P. R. China

Tel.: 86-22-60600045, Fax: 86-22-60602298. E-mail: minw@tust.edu.cn

Supplementary information

Supplementary information accompanies this paper at http://www.nature.com/srep

Data access: All of the data obtained by 454-sequencing were deposited in the NCBI Sequence Read Archive under accession number SRA182842. DNA sequences obtained from DGGE were deposited in the GenBank database under accession numbers KC961607 to KC961621.

Table S1 Data on 454-sequencing and α diversity metrics of the bacterial community in the whole fermentation of SAV

Samples[a]	No. of reads	No. of effective reads	OTUs	Sobs[b]	Chao[b]	ACE[b]	Shannon[b]	Simpson[b]
Daqu	17,980	5,680	118	118	224.000	274.707	2.327	0.182
AF1d	20,325	12,290	85	85	257.250	411.538	0.918	0.664
AF4d	73,338	48,944	134	134	301.647	508.238	1.028	0.481
AF7d	54,717	35,176	158	158	294.714	474.744	1.450	0.318
AF10d	89,585	55,129	135	135	335.200	623.921	1.397	0.315
AF12d	45,603	30,423	85	85	200.909	423.991	0.989	0.478
AAF0d	31,238	16,063	135	135	235.800	283.844	1.261	0.481
AAF1d	28,066	16,131	114	114	189.316	237.975	1.138	0.464
AAF3d	41,617	27,528	94	94	225.615	334.046	0.964	0.514
AAF5d	46,750	34,307	63	63	126.909	238.989	0.842	0.476
AAF7d	28,004	15,455	45	45	85.625	126.977	0.807	0.484

[a] Daqu represented sample used for starch saccharification, AF1d – AF12d represented samples in 1 d to 12 d of alcoholic fermentation, and AAF0d – AAF7d represent samples in 0 d to 7 d of acetic acid fermentation.

[b] α diversity indices in different samples.

Table S2 Phylogenetic identification of fungal community during the whole fermentation of SAV

[a]Band	Daqu			Alcohol fermentation			Acetic acid fermentation		
	Species	Similarity	Accession	Species	Similarity	Accession	Species	Similarity	Accession
1	*Saccharomycopsis fibuligera*	100%	AB550105	*Saccharomycopsis fibuligera*	100%	AB196495	*Saccharomycopsis fibuligera*	100%	AB550105
2	*Saccharomycopsis fibuligera*	100%	U09238	*Saccharomyces cerevisiae*	99%	EU327093	*Cladosporium cladosporioides*	98%	JX481886
3	*Eurotium intermedium*	99%	EF652068	*Saccharomyces cerevisiae*	98%	EU441887	*Alternaria alternate*	100%	JQ761707
4	*Monascus ruber*	99%	JN940513	*Saccharomyces cerevisiae*	100%	EU441887	*Saccharomyces cerevisiae*	100%	EU441887
5	*Pichia kudriavzevii*	100%	JX183970	*Saccharomyces cerevisiae*	99%	EU441887			
6				*Aspergillus niger*	100%	JX535499			

[a]Bands represent the number of bands in Fig. S1.

Table S3 Diversity metrics of fungal community in the whole fermentation of SAV

Samples[a]	Shannon	Simpson	Pielou
Daqu	2.90	0.06	0.99
AF1d	1.10	0.33	1.00
AF4d	1.08	0.34	0.98
AF7d	1.10	0.33	1.00
AF10d	1.10	0.33	1.00
AF12d	1.10	0.33	1.00
AAF0d	1.34	0.28	0.96
AAF1d	1.34	0.27	0.97
AAF3d	1.31	0.29	0.94
AAF5d	1.34	0.27	0.97
AAF7d	1.29	0.30	0.93

[a] Daqu represented specimen used for starch saccharification, AF1d - AF12d represented specimens in 1 day to 12 days of alcoholic fermentation, and AAF0d - AAF7d represented specimens in 0 day to 7 days of acetic acid fermentation.

Table S4. Relative concentrations of metabolites identified by GC-MS during the whole fermentation of SAV

No.	Compounds	Alcohol fermentation					Acetic acid fermentation				
		1d	3d	5d	10d	13d	0d	1d	3d	5d	7d
1	Alanine	0.345±0.124	0.631±0.107	0.191±0.092	1.599±0.491	0.916±0.197	0.512±0.139	0.569±0.153	0	0.924±0.212	0.143±0.086
2	Valine	0.137±0.083	0.411±0.096	0	0.387±0.121	0.454±0.085	0.273±0.064	0.201±0.106	0.074±0.036	0.391±0.138	0
3	Glycine	0.346±0.166	2.062±0.955	0	1.792±0.933	1.331±0.915	3.415±1.542	0.238±0.125	0.056±0.037	0.431±0.135	0.074±0.033
4	Leucine	0.158±0.106	1.092±0.907	0	0.662±0.225	0.532±0.185	0.413±0.158	0.191±0.094	0	0.492±0.202	0.039±0.018
5	Proline	0	0.724±0.248	0	0.306±0.154	0.316±0.125	0.439±0.163	0.085±0.056	0	0.931±0.162	0
6	Isoleucine	0	0	0	0.493±0.183	0.364±0.131	0.119±0.087	0	0	0.196±0.055	0
7	Serine	0.210±0.162	0.847±0.141	0	0.491±0.124	0.606±0.253	0.309±0.159	0.134±0.073	0	0.391±0.135	0.073±0.032
8	Threonine	0	0	0	0.881±0.278	0.832±0.228	0	0.174±0.0934	0.073±0.022	0.301±0.145	0
9	Aspartic acid	0.091±0.046	1.117±0.741	0	0	0	0.589±0.143	0.096±0.079	0	0.451±0.158	0
10	Lysine	0	0	0	0.729±0.398	0	0	0	0.047±0.035	0	0
11	Methionine	0	0	0	0	0	0	0	0	0.053±0.038	0
12	Ornithine	0	1.479±0.923	0	0	0	0	0	0	0.171±0.085	0
13	Acetic acid	0.096±0.056	0.259±0.137	0.173±0.103	0.173±0.132	0.128±0.084	0	0.039±0.012	0	0.092±0.053	0.081±0.022
14	Lactic acid	0	0	0.783±0.205	0	0	0.276±0.125	0	0.286±0.163	0	0
15	Propanoic acid	0.052±0.015	0.164±0.153	0.231±0.117	0.242±0.132	0.216±0.173	0.028±0.026	0.033±0.017	0.017±0.0012	0.035±0.019	0.008±0.0025
16	Propanedioic acid	0	0	0.226±0.125	0.134±0.095	0.055±0.43	0	0	0	0.024±0.015	0
17	Butanoic acid	0.035±0.016	0.233±0.179	0.203±0.138	1.704±0.995	0.526±0.205	0	0	0	0	0
18	Butanedioic acid	1.047±0.704	2.995±1.105	3.398±1.324	2.313±0.912	2.469±1.042	0.375±0.184	0.241±0.143	0.184±0.126	0.277±0.055	0.328±0.124
19	N-Dimethylglycine	0	8.938±2.781	0	0	0	0.085±0.039	0.079±0.014	0	0.081±0.029	0
20	2-Hydroxyiso	0	0.307±0.178	0.509±0.135	0.541±0.283	0.438±0.141	0.076±0.044	0.131±0.078	0.141±0.075	0.193±0.125	0.207±0.141

	caproic acid										
21	Malic acid	0	0.107±0.058	0	0	0	1.062±0.813	0.491±0.163	0.338±0.139	0.579±0.273	0.542±0.138
22	Pentanoic acid	0	0.144±0.087	0	0	0.091±0.041	0	0	0	0.119±0.073	0
23	Pentanedioic acid	0.156±0.093	1.683±0.462	0	0.711±0.257	0.669±0.235	0.115±0.073	0.069±0.024	0	0.112±0.069	0
24	Butenedioic acid	0	0	0	0	0	0	0	0	0.018±0.011	0
25	Benzenepropanoic acid	0	0.987±0.237	0	1.036±0.537	1.031±0.393	0.227±0.108	0.296±0.094	0.288±0.118	0.495±0. 143	0.527±0.177
26	Benzeneacetic acid	0.107±0.082	0.406±0.149	0.049±0.021	0	0	0	0	0	0	0
27	Benzoic acid	0.677±0.258	5.701±2.959	0	2.041±1.059	2.207±0.656	0.031±0.015	2.243±0.964	1.587±0.957	3.439±1.348	0.531±0.204
28	1,2,3-Propanetricarboxylic acid	0	0	0	0	0	2.053±0.918	0.022±0.016	0	0.061±0.023	0
29	Vanillylpropionic acid	0	0	0	0	0.329±0.158	0.061±0.031	0	0.024±0.019	0	0
30	Ferulic acid	0	0.019±0.016	0	0	0	0	0	0	0.037±0.016	0
31	Tartaric acid	0	0	0	0	0	0	0.085±0.043	0	0	0.042±0.028
	Fatty acids										
32	1-Benzazirene-1-carboxylic acid	0	0	0	0	0	0	0.017±0.013	0.013±0.012	0.042±0.028	0.031±0.019
33	n-Pentadecanoic acid	0.012±0.009	0	0.158±0.129	0.163±0.089	0	0	0	0	0	0
34	Hydrocinnamic acid	0	0.231±0.109	0	0	0.202±0.173	0	0	0	0	0
35	Hexadecanoic acid	0.188±0.109	0.379±0.153	0.162±0.105	0	0	0.383±0.179	0.312±0.205	0.135±0.095	0.259±0.171	0.061±0.045

36	Octadecanoic acid	0.086±0.067	0.309±0.108	0	0	0	0.336±0.168	0.147±0.137	0.164±0.289	0.319±0.144	0.183±0.139
37	Fructose	4.431±1.788	15.366±4.827	10.689±3.431	5.819±2.115	9.124±3.049	15.453±5.163	6.353±2.658	2.675±1.816	1.616±1.011	1.895±1.573
38	Fucose	0	0	0	0	0	0	0	0.192±0.124	0	0.334±0.133
39	Arabinofuranose	0	0.207±0.126	0.070±0.056	0.087±0.035	0.072±0.055	0	0	0.060±0.033	0.041±0.032	0.047±0.014
40	Sedoheptulose	2.845±0.786	10.935±2.923	5.905±1.887	0.052±0.044	0	0	0	0	0	0
41	Xylulose	0.897±0.117	0	0.102±0.067	0	0	0	0	0	0	0
42	Xylose	4.088±1.947	0.808±0.283	0.579±0.312	3.637±1.067	1.162±0.959	1.071±0.602	2.544±1.083	0.934±0.386	6.687±2.548	2.215±0.816
43	Xylopyranose	0.071±0.042	0.119±0.103	0.551±0.169	3.965±1.429	6.663±2.694	0	0	2.274±1.531	0.659±0.443	0.485±0.218
44	Xylitol	0	0.272±0.171	0	0	0.026±0.017	0.104±0.045	0.947±0.207	0.055±0.029	0.447±0.282	0.466±0.143
45	Xylonic acid	0	0.291±0.124	0.251±0.109	0.233±0.095	0.239±0.104	0	0	0	0.053±0.035	0.064±0.034
46	Ribose	7.224±1.461	0	1.401±0.606	0.768±0.357	0	0.063±0.058	0.261±0.175	0	0.662±0.267	0.491±0.189
47	Galactose	9.154±3.423	7.703±2.493	2.325±0.843	0.355±0.178	0.438±0.145	1.252±0.468	1.688±0.763	1.907±0.646	4.154±1.072	5.715±1.349
48	Galactopyranose	0.408±0.276	0.404±0.146	0.393±0.337	0	0.297±0.188	1.209±0.756	0.215±0.152	0	0.061±0.032	0.411±0.136
49	Galactofuranose	1.033±0.559	0.275±0.165	0.185±0.159	0.073±0.068	0	0	0	0.056±0.023	0	0
50	Galactopyranoside	0	0.167±0.122	0	0	0	0	0	0.059±0.039	0	0.004±0.003
51	Galactonic acid	0	0	0	0.191±0.045	0.356±0.155	0	0	0	0	0
52	Dulcitol	0.690±0.189	1.957±0.923	1.985±0.751	1.161±0.732	1.236±0.909	0.100±0.048	0.122±0.074	0.105±0.102	0.202±0.127	0.166±0.098
53	Mannose	25.106±6.528	8.058±2.683	3.332±1.469	0.851±0.398	0.614±0.282	15.405±3.698	11.248±2.435	7.451±1.295	0.577±0.104	1.503±0.817
54	Mannonic acid	0	0	0	0.055±0.048	0	0	0	0	0	0.505±0.174
55	Mannopyranose	0	0.064±0.019	0	0	0	0	0	0	0	0.294±0.125
56	Mannopyranoside	0.098±0.046	0	0.031±0.014	0	0	0	0	0	0.016±0.012	0.062±0.042

57	Lyxose	0	0	0.237±0.134	0.289±0.189	1.559±0.826	0.131±0.039	0.024±0.017	0	0.361±0.105	0
58	Lyxopyranose	0	0.145±0.991	0.266±27945	0.535±0.149	0.044±0.024	0	0.221±0.146	0	0	0
59	Glucopyranose	7.555±2.257	5.878±1.751	1.559±0.722	0	0.126±0.113	1.004±0.804	0.466±0.294	0.228±0.127	0.397±0.166	1.032±0.635
60	Glucopyranoside	0.128±0.083	0	0	0	0	0	0	0.160±0.102	0.046±0.017	0
61	Glucose	136.039±17.208	56.882±12.397	9.703±4.863	1.021±0.698	0.326±0.144	24.898±7.211	9.277±2.467	2.587±1.356	17.645±6.861	21.401±6.392
62	Gluconic acid	0.403±0.283	0.654±0.235	0.035±0.017	0.171±0.135	0.442±0.121	0.601±0.134	0.564±0.258	0	0.915±0.327	0.355±0.187
63	Talose	9.312±2.647	0	0.341±0.192	0	0	0.724±0.248	0	0	0	1.215±0.636
64	Melibiose	0	0.112±0.078	0	0	0.131±0.066	0	0	0	0.177±0.104	0
65	Sorbopyranose	0	0	0	0	0	0	0	0	0	0.069±0.023
66	Maltose	0	0.232±0.149	0	0	0	0.332±0.169	0	0	0	0
67	Xylopyranoside	0	0	0	0	0	0	0.041±0.027	0.045±0.018	0	0
68	Erythro-Pentitol	0	0.211±0.137	0.117±0.083	0.108±0.659	0.131±0.059	0	0	0	0	0
69	Arabitol	3.202±0.968	15.756±3.028	14.624±2.838	13.348±2.611	10.943±1.492	1.001±0.866	1.012±0.648	0.716±0.268	2.515±0.715	1.751±0.621
70	Arabinitol	0	0.080±0.025	0.084±0.032	0	0	0.038±0.033	0	0	0	0.123±0.049
71	Ribo-Hexitol	0	0.131±0.1272	0.159±0.165	0.063±0.041	0.074±0.051	0	0	0	0	0
72	Mannitol	2.207±0.777	5.273±1.347	3.344±0.826	0.349±0.198	0.085±0.233	0.683±0.262	0.782±0.208	0.355±0.166	1.321±0.418	0.676±0.106
73	glucitol	2.041±0.779	1.809±0.956	1.861±0.818	1.071±0.432	1.344±0.641	1.059±0.779	0.232±0.176	0.323±0.167	0.482±0.141	0.666±0.236
74	Ribitol	0	0.067±0.045	0.057±0.016	0	0.047±0.028	0.252±0.098	0	0	0.216±0.148	0.202±0.072
75	Adonitol	2.760±0.443	25.163±8.437	23.768±5.312	14.702±2.758	9.382±1.507	0	0	0	0	0.138±0.115
76	Inositol	0.111±0.079	0.257±0.108	0.202±0.105	0.229±0.176	0.141±0.094	0.027±0.015	0.046±0.018	0.014±0.005	0.015±0.014	0.024±0.025
77	Myo-Inositol	9.148±1.701	21.026±3.048	21.212±3.221	17.323±2.346	13.293±1.607	2.789±1.142	2.901±0.851	3.188±1.038	5.238±1.882	5.548±2.044
78	Gulonic acid	0	0.012±0.002	0	0.223±0.143	0	0.348±0.179	0	0	0	0.463±0.115

79	2-Keto-l-gluconic acid	0	0	0	0	0.0893831	0	0	0	0.099897876	0.0975219
80	Threonic acid	0	0.799±0.454	0	0.242±0.095	0.351±0.106	0.073±0.046	0.052±0.024	0.019±0.013	0.064±0.039	0
81	2-O-Glycerol-.alpha.-d-galactopyranoside	1.142±0.727	0.574±1.658	0.077±0.023	0.326±0.142	0.066±0.021	0.168±0.083	0.067±0.038	0	0.222±0.144	0.311±0.135
82	Arabinonic acid	0	0	0.238±0.135	0.317±0.166	0.148±0.042	0	0	0	0.024±0.012	0
83	Ribonic acid	0.059±0.022	0.621±0.151	0	0	0	0	1.907±0.435	0	0	0
84	Glutamine	0	0	0	0	0	0.588±0.199	0	4.251±1.235	0.166±0.102	0
85	Cadaverine	0.079±0037	0	0	0	1.566±0.383	0	0	0	0	0
86	1,4-Butanediamine	0	0	0	0	3.589±0.559	0	0	0	0	0
87	carbamic acid ethyl ester	0.544±0.327	0	0	0	0	0	0.188±0.103	0	0	0

The values are calculated by comparing their peak areas with those of the internal standard compound. The quantitative data are the mean values of triplicate measurements. All values are presented as the mean±SD.

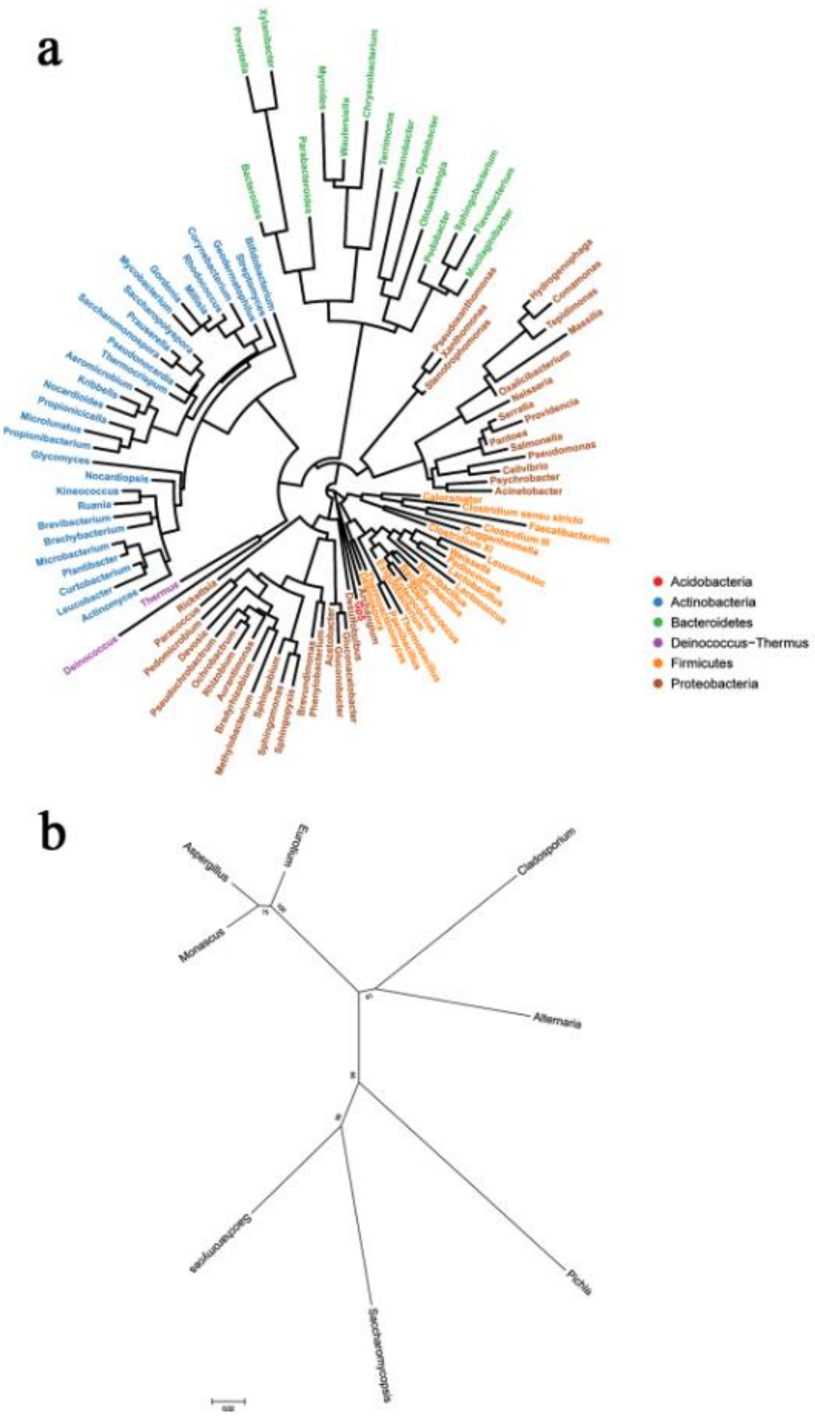

Figure S1. Phylogenetic tree of bacteria (a) and fungi (b) in SAV.

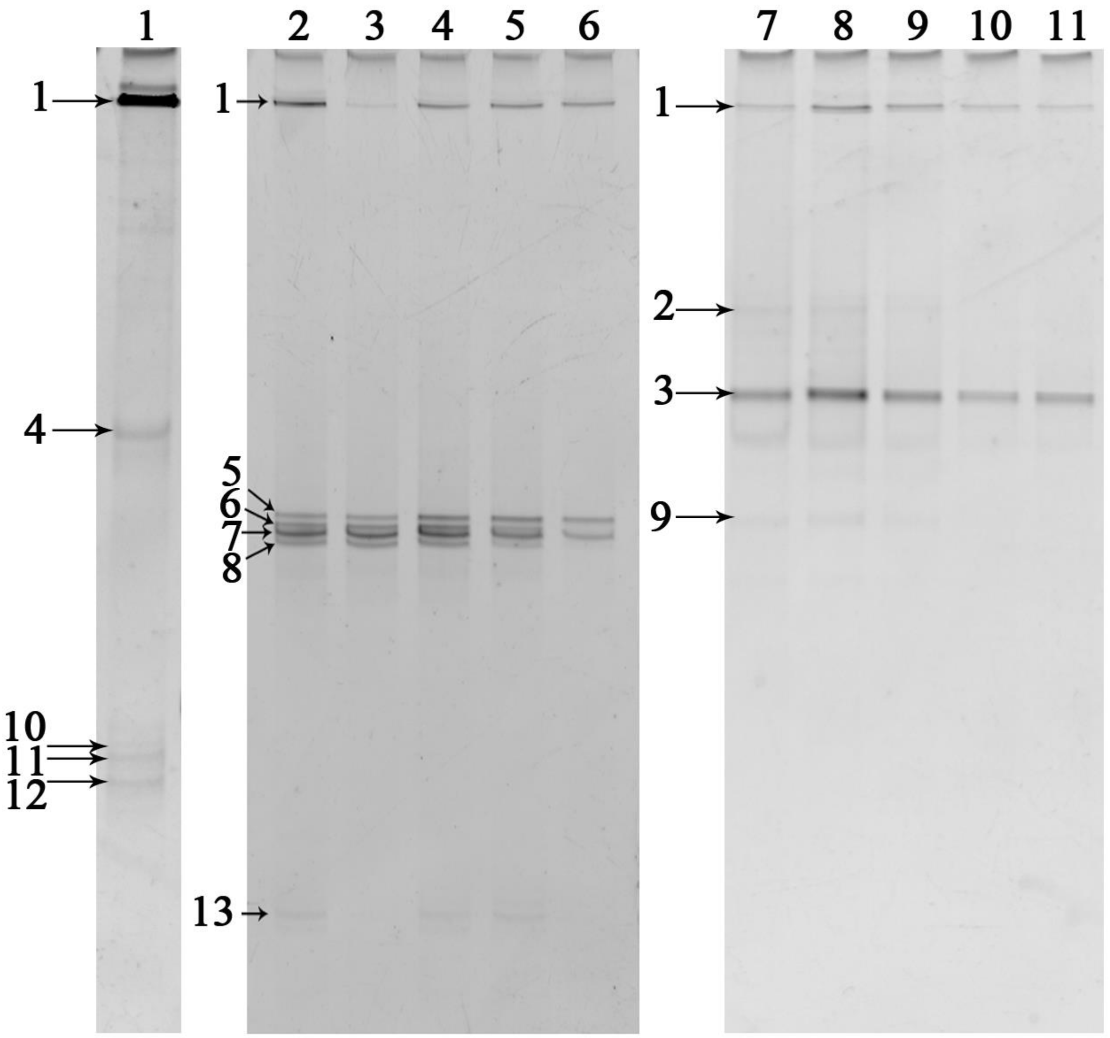

Figure S2. DGGE fingerprint of the fungal community during the whole fermentation of Shanxi aged vinegar. Lane 1 represents the *daqu* specimen. Lanes 2 to 6 represent jiulao specimens at 1, 4, 7, 10, and 12 days of alcoholic fermentation. Lanes 7 to 11 represent cupei specimens at 0, 1, 4, 5, and 7 days of acetic acid fermentation.
